# Supplementary material for: Complete Mitochondrial Genome for Lucilia cuprina dorsalis (Diptera: Calliphoridae) from the Northern Territory, Australia
Source: Genes (Basel). 2024 Apr 18;15(4):506. doi: 10.3390/genes15040506 (PMC11050061; doi:10.3390/genes15040506)
Supplement: Supplementary file 1 [file genes-15-00506-s001.zip › Table S1.pdf]

Table S1
Details on Dipteran mitochondrial (mt) genomic sequences and collection locations used for the phylogenetic analysis in the present study.

**Species**	**Family**	**Accession number**	**Length (bp)**	**Place of origin**	**Reference**
Calliphora chinghaiensis	Calliphoridae	NC_029215	15269	China	(Chen et al., 2016)
Calliphora nigribarbis	Calliphoridae	MK893470	16279	South Korea	(Karagozlu et al., 2019)
Calliphora vicina	Calliphoridae	NC_019639	16112	France (Voucher-DI242)	(Nelson et al., 2012)
Calliphora vomitoria	Calliphoridae	NC_028411	16134	China	(Ren et al., 2016)
Chrysomya megacephala	Calliphoridae	AJ426041	15831	Calicut, Kerala State, India	(Stevens et al., 2008)
Chrysomya pinguis	Calliphoridae	NC_025338	15838	China	Yan et al., 2016
Chrysomya putoria	Calliphoridae	AF352790	15837	Brazil	(Junqueira et al., 2004)
Chrysomya rufifacies strain DI215	Calliphoridae	JX913740	15412	El Questro Resort, WA, Australia (Voucher-D215)	(Nelson et al., 2012)
Chrysomya villeneuvi	Calliphoridae	MW592365	15623	China	(Guo and Zhang) (unpublished)
Cochliomyia hominivorax	Calliphoridae	AF260826	16022	Brazil	(Lessinger and Azeredo-Espin, 2000)
Dermatobia hominis	Oestridae	NC_006378	16360	Brazil	(Azeredo-Espin et al., 2004)
Exorista japonica	Tachinidae	NC_044409	17663	South Korea	(Seo et al., 2019)
Exorista sorbillans	Tachinidae	NC_014704	14960	China	(Shao et al., 2012)
Haematobia irritans irritans	Muscidae	DQ029097	16078	Brazil	(Oliveira et al., 2008)
Hemipyrellia ligurriens	Calliphoridae	NC_019638	15938	University of Queensland campus, St Lucia,	(Nelson et al., 2012)

				Brisbane, Qld, Australia	
Hypoderma lineatum	Oestridae	NC_013932	16354	Italy	(Weigl et al., 2010)
Hypoderma sinense	Oestridae	NC_071819	16296	China	(Tang et al.) (unpublished)
Lucilia caesar isolate C2	Calliphoridae	NC_028057	15954	UK	(Schoofs et al.) (unpublished)
Lucilia caesar isolate C3	Calliphoridae	KM657112	15957	UK	(Schoofs et al.) (unpublished)
Lucilia coeruleiviridis	Calliphoridae	NC_029486	14989	USA	(Junqueira et al., 2016)
Lucilia cuprina	Calliphoridae	KT272779	14943	Brazil	(Junqueira et al., 2016)
Lucilia cuprina cuprina QLD, Australia	Calliphoridae	MW255538	15952	QLD, Australia	(Kapoor et al., 2023)
Lucilia cuprina dorsalis NSW, Australia	Calliphoridae	MW255537	15941	NSW, Australia	(Kapoor et al., 2023)
Lucilia cuprina dorsalis VIC, Australia	Calliphoridae	MW255536	15941	VIC, Australia	(Kapoor et al., 2023)
Lucilia cuprina dorsalis WA, Australia	Calliphoridae	MW255539	15944	WA, Australia	(Kapoor et al., 2023)
Lucilia cuprina dorsalis NT, Australia	Calliphoridae	PP297113	15943	NT, Australia	Present study
Lucilia cuprina strain DI190.1 Melbourne, Australia	Calliphoridae	JX913744	15952	University of Melbourne colony; (P. Batterham) (Voucher - DI190.1)	(Nelson et al., 2012)
Lucilia cuprina strain DI190.2 Melbourne, Australia	Calliphoridae	JX913745	15950	University of Melbourne colony; (P. Batterham) (Voucher - DI190.2)	(Nelson et al., 2012)

Lucilia cuprina strain DI190.3 Melbourne, Australia	Calliphoridae	JX913746	15952	University of Melbourne colony; (P. Batterham) (Voucher - DI190.3)	(Nelson et al., 2012)
Lucilia cuprina strain DI190.4 Melbourne, Australia	Calliphoridae	JX913747	15943	University of Melbourne colony; (P. Batterham) (Voucher - DI190.4)	(Nelson et al., 2012)
Lucilia cuprina strain DI190.5 Melbourne, Australia	Calliphoridae	JX913748	15946	University of Melbourne colony; (P. Batterham) (Voucher - DI190.5)	(Nelson et al., 2012)
Lucilia cuprina strain DI213.1 QLD, Australia	Calliphoridae	JX913749	15348	Petrie Terrace, Brisbane, QLD (Voucher-DI213.1)	(Nelson et al., 2012)
Lucilia cuprina strain DI213.2 QLD, Australia	Calliphoridae	JX913750	15310	Petrie Terrace, Brisbane, QLD (Voucher-DI213.2)	(Nelson et al., 2012)
Lucilia cuprina strain DI213.3 QLD, Australia	Calliphoridae	JX913751	15289	Petrie Terrace, Brisbane, QLD (Voucher-DI213.3)	(Nelson et al., 2012)
Lucilia cuprina strain DI213.4 QLD, Australia	Calliphoridae	JX913752	15268	Petrie Terrace, Brisbane, QLD (Voucher-DI213.4)	(Nelson et al., 2012)
Lucilia cuprina strain DI213.5 QLD, Australia	Calliphoridae	JX913753	15226	Petrie Terrace, Brisbane, QLD (Voucher-DI213.5)	(Nelson et al., 2012)
Lucilia hainanensis	Calliphoridae	MW592363	15319	China	(Guo nd Zhang) (unpublished)
Lucilia illustris	Calliphoridae	KT272845	14875	USA	(Junqueira et al., 2016)
Lucilia illustris isolate 1sp10	Calliphoridae	NC_028056	15954	UK	(Schoofs et al.) (unpublished)
Lucilia illustris isolate 1sp11	Calliphoridae	KM657110	15956	UK	(Schoofs et al.) (unpublished)

Lucilia papuensis isolate C44	Calliphoridae	MH540746	15884	China	(Ma and Huang) (unpublished)
Lucilia papuensis voucher CSU19111932	Calliphoridae	NC_053672	15323	China	(Guo and Ren) (unpublished)
Lucilia porphyrina	Calliphoridae	NC_019637	15877	University of Queensland campus, St Lucia, Brisbane, QLD, Australia (Voucher - DI211)	(Nelson et al., 2012)
Lucilia sericata	Calliphoridae	KT272854	15092	USA	(Junqueira et al., 2016)
Lucilia sericata strain DI220 QLD, Australia	Calliphoridae	JX913755	15300	Queensland Department of Primary Industries and Fisheries Agricultural Research Institute lab strain (Voucher-DI220)	(Nelson et al., 2012)
Lucilia sericata strain DI245 WA, Australia	Calliphoridae	JX913756	15214	Perth, WA, Australia (Voucher-DI245)	(Nelson et al., 2012)
Lucilia sericata strain DI246 ACT, Australia	Calliphoridae	JX913754	15243	Canberra, ACT, Australia (Voucher-DI246)	(Nelson et al., 2012)
Lucilia sericata strain DI257 Utah, USA	Calliphoridae	JX913757	15380	Brigham Young University campus, Provo, UT, USA (Voucher-DI257)	(Nelson et al., 2012)

Lucilia sericata TAS, Australia	Calliphoridae	MW255540	15946	TAS, Australia	(Kapoor et al., 2023)
Lucilia sericata UK	Calliphoridae	AJ422212	15945	UK	(Stevens et al., 2008)
Lucilia shenyangensis	Calliphoridae	NC_059913	14989	China	(Chen) (unpublished)
Rutilia goerlingiana	Tachinidae	NC_019640	15331	Mary River Roadhouse, Burrundie, NT, Australia	(Nelson et al., 2012)
Sarcophaga brevicornis	Sacrophagidae	NC_047404	15152	China	(Zhang et al., 2019)
Sarcophaga impatiens	Sacrophagidae	NC_017605	15169	University of Wollongong, NSW, Australia	(Nelson et al., 2012)
Sarcophaga kanoi	Sacrophagidae	NC_051537	15319	China	(She et al., 2019)
Sarcophaga tuberosa	Sacrophagidae	MK820723	15173	China	(Kai et al., 2019)
