# Supplementary material for: Complete Mitochondrial Genome for Lucilia cuprina dorsalis (Diptera: Calliphoridae) from the Northern Territory, Australia
Source: Genes (Basel). 2024 Apr 18;15(4):506. doi: 10.3390/genes15040506 (PMC11050061; doi:10.3390/genes15040506)
Supplement: Supplementary file 1 [file genes-15-00506-s001.zip › Table S2.pdf]

Table S2
Mitochondrial (mt) genome structure of *Lucilia cuprina dorsalis* from the Northern Territory in Australia.

		***L. c. dorsalis* (Northern Territory, Australia)**			
**Genes**	**Location**	**Transcription direction**	**Length (bp)**	**Start codon**	**Stop codon**
rrnS	14612-14873	reverse	787		
trnV(tac)	14542-14613	reverse	72		
rrnL	13193-14485	reverse	1293		
trnL1(tag)	13149-13213	reverse	65		
nad1	12200-13218	reverse	939	ATA	TAA
trnS2(tga)	12116-12183	forward	68		
cob	10981-12117	forward	1137	ATG	TAA
nad6	10487-10981	forward	495	ATT	TAA
trnP(tgg)	10389-10454	reverse	66		
trnT(tgt)	10324-10388	forward	65		
nad4l	10025-10321	reverse	297	ATG	TAA
nad4	8631-9965	reverse	1335	ATG	TAA
trnH(gtg)	8628-8692	forward	65		
nad5	6890-8603	reverse	1714	ATT	Incomplete
trnF(gaa)	6826-6892	reverse	67		
trnE(ttc)	6740-6807	forward	68		
trnS1(gct)	6670-6737	forward	68		
trnN(gtt)	6604-6669	forward	66		
trnR(tcg)	6541-6603	forward	63		
trnA(tgc)	6477-6541	forward	65		

nad3	6121-6474	forward	354	ATT	TAA
trnG(tcc)	6056-6120	forward	65		
cox3	5260-6048	forward	789	ATG	TAA
atp6	4583-5260	forward	678	ATG	TAA
atp8	4425-4589	forward	165	ATT	TAA
trnD(gtc)	4358-4424	forward	67		
trnK(ctt)	4288-4358	forward	71		
cox2	3600-4284	forward	685	ATG	Incomplete
trnL2(taa)	3528-3593	forward	66		
cox1	1994-3532	forward	1539	TCG	TAA
trnY(gta)	1929-1995	reverse	67		
trnC(gca)	1863-1926	reverse	64		
trnW(tca)	1803-1870	forward	68		
nad2	808-1803	forward	996	ATT	TAA
trnM(cat)	718-786	forward	69		
trnQ(ttg)	644-712	reverse	69		
trnI(gat)	581-646	forward	66		
