# Supplementary material for: Complete Mitochondrial Genome for Lucilia cuprina dorsalis (Diptera: Calliphoridae) from the Northern Territory, Australia
Source: Genes (Basel). 2024 Apr 18;15(4):506. doi: 10.3390/genes15040506 (PMC11050061; doi:10.3390/genes15040506)
Supplement: Supplementary file 1 [file genes-15-00506-s001.zip › Table S3.pdf]

Table S3
Nucleotide polymorphisms in the mitochondrial (mt) genome of *Lucilia cuprina dorsalis* collected from the Northern Territory in Australia.

**Position**	**Polymorphism**	**Reference**	**Variant**	**Gene**
485	SNP (transition)	A	G	*nad2*
695	SNP (transition)	A	G	*nad2*
752	SNP (transition)	A	G	*nad2*
984	SNP (transversion)	A	T	*nad2*
1079	SNP (transition)	G	A	*nad2*
1190	SNP (transition)	T	C	*nad2*
1211	SNP (transition)	C	T	*nad2*
1524	SNP (transition)	C	T	*nad2*
1692	SNP (transversion)	T	A	*nad2*
1713	SNP (transition)	C	T	*cox2*
1878	SNP (transition)	C	T	*cox2*
1908	SNP (transition)	T	C	*cox2*
2127	SNP (transition)	C	T	*cox2*
2145	SNP (transition)	C	T	*cox2*
2157	SNP (transition)	T	C	*cox2*
2184	SNP (transition)	T	C	*cox2*
7960	SNP (transition)	C	T	*nad5*
9192	SNP (transversion)	T	A	*nad4*
9216	SNP (transition)	T	C	*nad4*
10680	SNP (transition)	C	T	*cob*
11124	SNP (transversion)	C	A	*cob*
11699	SNP (transition)	T	C	*nad1*
11765	SNP (transition)	C	T	*nad1*
11798	SNP (transition)	C	T	*nad1*
11852	SNP (transversion)	A	T	*nad1*
11972	SNP (transition)	T	C	*nad1*
15938	SNP (transversion)	T	A	
15939	SNP (transversion)	T	A	
